# Persistent Left Superior Vena Cava (PLSVC) with a Connection to the Azygos System: Case Report and Clinical Implications

**DOI:** 10.1155/2019/5390272

**Published:** 2019-08-05

**Authors:** Alastair E. Moody, Catriona E. Moody, Bryce D. Beutler, Micaela M. Koci

**Affiliations:** ^1^University of Utah, Department of Anesthesia, 30 N 1900 E, Room 3C444, Salt Lake City, UT 84132, USA; ^2^University of Nevada, Reno School of Medicine, Department of Internal Medicine, 1155 Mill Street, W-11, Reno, NV 89502, USA; ^3^University of Nevada, Reno School of Medicine, 1664 N. Virginia St., Reno, NV 89557, USA

## Abstract

Persistent left superior vena cava (PLSVC) is a rare anatomic variant that has a significant effect on the structure of the heart and venous system with clinical implications that are far-reaching. The presence of this variant is relevant to central venous catheter insertion, cardioverter-defibrillator placement, coronary artery bypass grafting, and numerous other medical procedures. In this report, we describe a rare case of PLSVC with a connection to the azygos system; notably, the vast majority of PLSVCs connect to the coronary sinus. We also discuss the anatomic and anesthetic considerations for individuals with this uncommon variant.

## 1. Introduction

Persistent left-sided superior vena cava (PLSVC) is an anatomic variant that has been reported to occur in 0.3% of the general population and in up to 4.3% of patients with other cardiac abnormalities [1, 2]. This variant has been known to the medical community for some time, with the first reported case in the literature dating back to the late 1800s [3]. Investigators have hypothesized that this anatomical variant is the result of aberrant growth from the left anterior cardinal vein and the left common cardinal vein during embryogenesis [4]. In most individuals, this abnormal growth pattern results in a left-sided superior vena cava connecting to a dilated coronary sinus in the heart.

Identification of PLSVC is critically important for central venous line placement as cannulation of the heart would be almost impossible from the left side. Over five million central lines are placed in the United States every year, and thus recognition of this anatomic variant can help improve outcomes for a significant portion of the population [5].

## 2. Case Presentation

A 37-year-old female with a history of fibromyalgia and poor venous access was admitted for port placement. She had chronic deep vein thromboses, including a celiac artery thrombosis, and associated embolic events which required long-term anticoagulation with warfarin. Multiple attempts were made to place both right and left subclavian ports, all of which were unsuccessful due to the inability to pass the wire into the superior vena cava. Consequently, the previous right subclavian port was exchanged.

**Figure 1:**
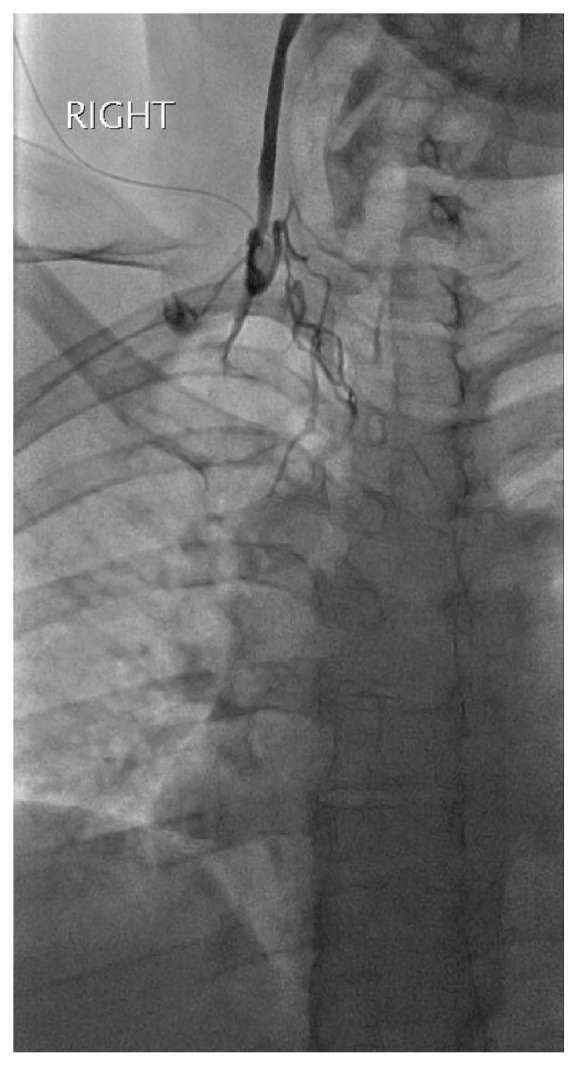
Right internal jugular venogram via micropuncture access revealing an occluded right internal jugular vein.

**Figure 2:**
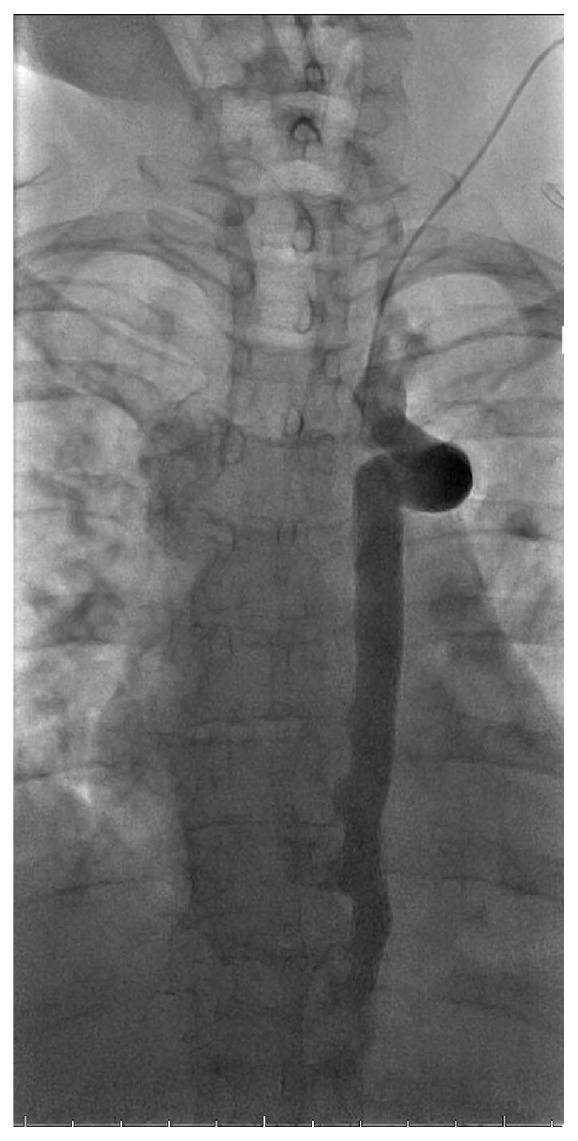
Left internal jugular venogram (early phase) demonstrating persistent left superior vena cava (PLSVC) with continuation into the hemiazygos vein.

**Figure 3:**
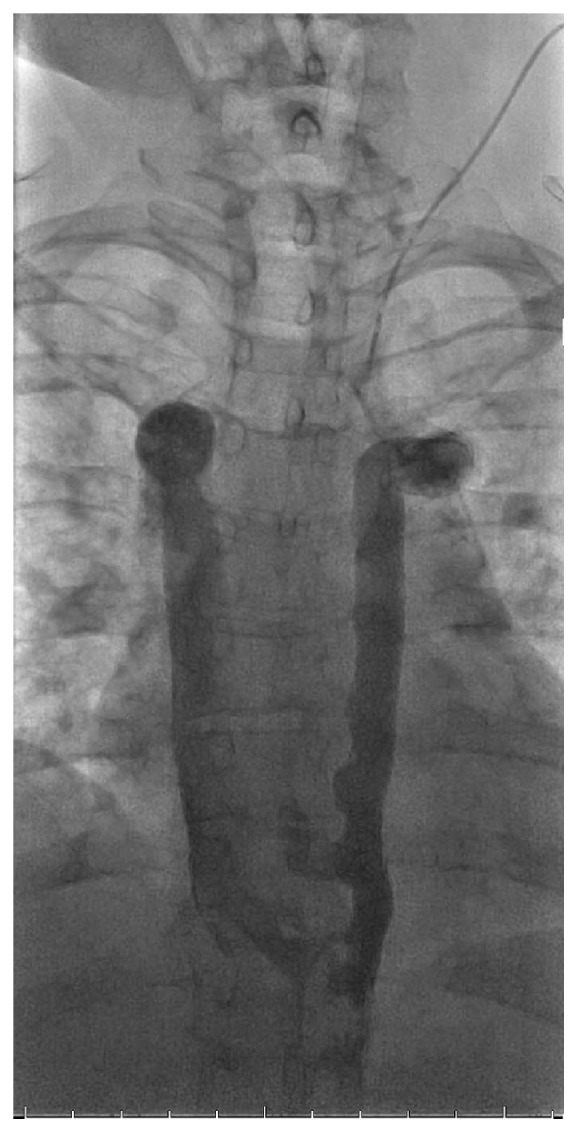
Left internal jugular venogram (mid phase) demonstrating PLSVC with continuation into the hemiazygos vein. Collaterals cross midline from left to right into the azygos vein at the T8 and T9 levels with subsequent drainage into the right superior vena cava (SVC).

**Figure 4:**
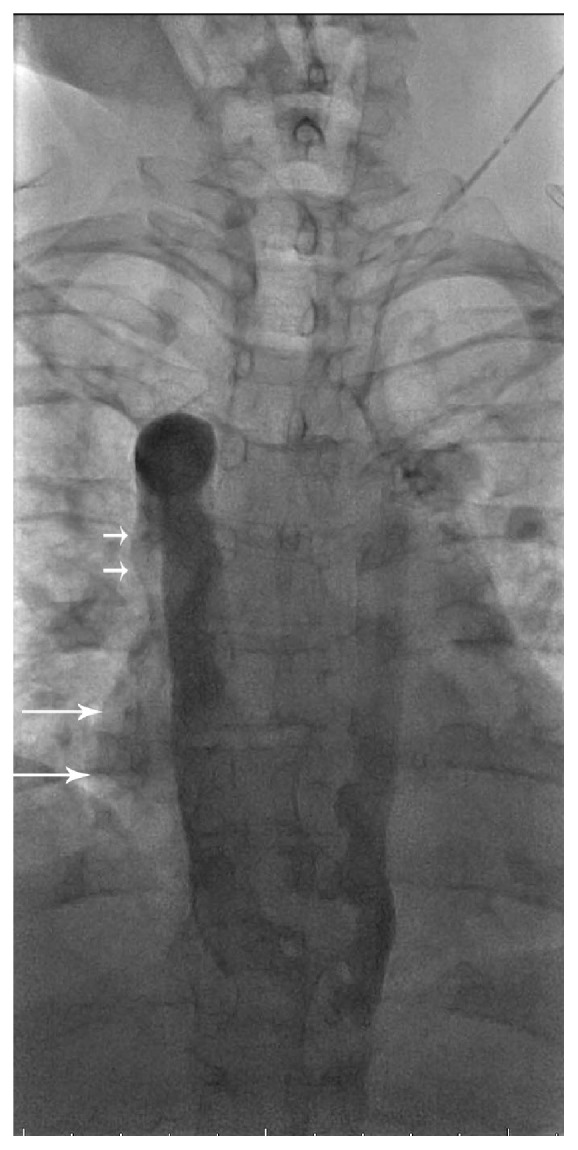
Left internal jugular venogram (late phase) demonstrating PLSVC with continuation into the hemiazygos vein. Collaterals cross midline from left to right into the azygos vein at the T8 and T9 levels with subsequent drainage into the right SVC. There is visualization of streaming of contrast outlining the lateral margin of the SVC (short arrows) and right atrium (long arrows).

Four weeks after the port was exchanged the patient complained of severe pain around the port site. The port was subsequently removed; a small seroma was found, but there were no signs of infection. Interventional radiology was consulted for further evaluation and port placement. Interventional radiology performed venograms of the right and left internal jugular veins as well as the right axillary and subclavian veins. The right venogram demonstrated occlusion of the right internal jugular vein (Figure 1). The left venogram revealed an atypical anatomic structure: the left brachiocephalic vein was absent and the left internal jugular vein drained into a left-sided superior vena cava (SVC) (Figure 2). This left-sided paramediastinal SVC descended in a course similar to an accessory hemiazygos vein, continuing into the hemiazygos vein with collaterals crossing midline from left to right into the azygos vein at the levels of T8 and T9 (Figures 3 and 4). The azygos vein, in turn, drained directly into the right SVC. Right axillary-subclavian venograms showed normal anatomy and patency of all vasculature. A port was placed in the right subclavian vein under ultrasound and fluoroscopic guidance. The patient was discharged without any complications.

## 3. Discussion

PLSVC occurs in approximately 0.3% of the general population, affecting nearly ten million individuals in the United States alone [1]. Early recognition of this anatomic variant can therefore improve outcomes for a significant number of patients who require central venous access. Notably, individuals with PLSVC are usually asymptomatic, and up to 80% of those with PLSVC exhibit normal right-sided vasculature [2]. Central venous catheterization is most commonly performed on the right side and thus providers often fail to identify PLSVC even in affected individuals undergoing port placement.

There are few reported cases of a duplicated left-sided SVC with a connection to the azygous system. In our patient, the PLSVC descended along the course of the accessory hemiazygos vein and crossed the midline at the T8-T9 level to connect to the azygos vein on the right side. After forming a connection with the azygos vein, the duplicated left-sided SVC followed its normal anatomical course back to the heart [6]. The brachiocephalic vein is absent; the left internal jugular vein drained directly into the PLSVC instead of following its normal course to the right heart.

The atypical anatomic variant described above creates a challenging scenario for the establishment of central venous access. It is significantly more difficult to access the heart as compared to individuals with the more common variant of PLSVC in which the vessel drains directly into the right atrium through the dilated coronary sinus. The prevalence of the variant observed in our patient is unknown. However, when thoracic venous abnormalities are identified, the most common variant is a PLSVC in the setting of a normal right-sided SVC. In 92% of individuals with PLSVC, the PLSVC drains into the right atrium through a dilated coronary sinus [7]. The remaining 8% of PLSVCs drain directly into the left atrium, leading to shunting and an increased risk of emboli upon placement of a central venous catheter. Our patient was unique in that the structure of her PLSVC was distinct from either of the previously recognized variants.

In our patient, left-sided central venous access was not possible due to the variation in the patient's vasculature. This rare variant may be recognized by failure to pass a guidewire, as in this case, or due to complications after attempting to establish access. Multiple attempts to place a left subclavian port in this patient were unsuccessful for this reason. In our patient, a venogram was required to diagnose the underlying abnormality.

The diagnosis of PLSVC can usually be established with contrast echocardiography, which shows dilation and opacification of the coronary sinus [8]. In our patient, however, the coronary sinus would have appeared normal due to the connection to the azygos system. It is conceivable that computed tomography (CT) or magnetic resonance imaging (MRI) of the chest would have revealed our patient's unique anatomic variant. Indeed, MRI provides excellent visualization of the mediastinal structures, including the vena cavae and coronary sinus. CT angiography with digital subtraction may also demonstrate PLSVC in individuals without coronary sinus dilation [9]. Cardiac catheterization is unnecessary for most patients with suspected PLSVC, but would likely show a step-down in oxygen saturation between the pulmonary veins and left atrium [10].

There are many clinical implications to consider when variant venous vasculature is discovered. In this patient, left-sided venous access was impossible. However, in other patients with more common variants of PLSVC, special consideration must be taken before attempting placement of Swan-Ganz catheters and implantable cardioverter-defibrillators. PLSVCs can also complicate cardiac surgery. Furthermore, PLSVCs are considered a relative contraindication to retrograde cardioplegia, as perfusion up the PLSVC can lead to impaired myocardial protection [11, 12].

Knowledge of the possible anatomical variations in the vascular system is important during placement of central venous catheters. Clinicians should consider these variants and the inherent associated risks when these variants are encountered.
